# Characterization of Structure and Antioxidant Activity of Polysaccharides From Sesame Seed Hull

**DOI:** 10.3389/fnut.2022.928972

**Published:** 2022-06-21

**Authors:** Run-Yang Zhang, Jing-Hao Gao, Yi-Lin Shi, Yi-Fei Lan, Hua-Min Liu, Wen-Xue Zhu, Xue-De Wang

**Affiliations:** Institute of Special Oilseed Processing and Technology, College of Food Science and Technology, Henan University of Technology, Zhengzhou, China

**Keywords:** sesame seed, hull, polysaccharides, chemical structure, antioxidant activity

## Abstract

Sesame seed hull is the major by-product of sesame seed processing and is rich in polysaccharides. In this work, sesame hull polysaccharides (SHP) were extracted by ultrasound-assisted alkali extraction methods with a yield of 6.49%. Three purified polysaccharide fractions were obtained after decolorization, deproteinization, and column chromatography. Then, their main composition and antioxidant activity were investigated. The dominant fraction was SHP-2 with a yield of 3.78%. It was composed of galacturonic acid (51.3%), glucuronic acid (13.8%), rhamnose (8.9%), glucose (8.4%), and others. The linkage types of SHP-2 have the α-D-Gal*p*A-(1,4)-linked, α-D-Glc*p*A-(1,2)-linked, β-T-D-Rha*p*-linked, β-D-Glc*p*-(1,6)-linked, β-T-D-Gal*p*-linked, α-L-Xyl*p*-(1,4)-linked, α-L-Ara*f*-(1,3,5)-linked, and β-D-Man*p*-(1,4)-linked. This study might provide some useful basic data for developing applications for sesame seed hull polysaccharides in the food and pharmaceutical industries.

## Introduction

Polysaccharide is a macromolecule found in almost all organisms. Structurally, polysaccharides are chains of monosaccharides connected by glycosidic linkages (1, 2). They have many functional and/or biological properties. The science of using polysaccharide-based substances in manufacturing health and biomedical products, cosmetics, human food, and animal feed is rapidly evolving (3–5). Rising demand is fueling the search for more and better sources of natural polysaccharides.

To meet this demand, particular attention has been paid to plant oil seeds, because the by-products of oil production are typically rich in polysaccharides (6). Sesame (*Sesamum indicum* L., Pedaliaceae) is a popular edible oil raw material in Asia and Africa (7). However, the oil derived from un-hulled sesame seeds is inferior due to the presence of bitter substances in the sesame hull, such as oxalic acid and tannin (8, 9). Therefore, dehulling is a critical operation during the manufacture of sesame-based products, such as sesame oil, sesame protein, and sweetened sesame paste (10, 11). By weight, the hull constitutes 17.0–19.0% (see section “Materials and Chemical Reagents”) of the whole sesame seed. Thus, during the dehulling process, a large amount of sesame hulls is produced as a production by-product. Currently, these hulls are usually simply discarded and not fully utilized, but it is rich in polysaccharides (42.7%) and contains high amounts of dietary fiber (25.6%) (12). Moreover, sesame hull was found to contain pectin with linear homogalacturonan as the main structure, with a yield of 9.72% and good free radical-scavenging ability (9). These characteristics suggest that sesame hulls might be a raw material of function polysaccharides and low-calorie foods. Nowadays, a wide range of polysaccharides has already found applications in human health, such as functional food and bioactive compounds. In addition, these polysaccharides generally have excellent antioxidant properties. According to epidemiological studies, natural antioxidants in the diet give rise to a lower probability of cardiovascular disease and cancer (2). Obviously, to promote the usage of waste sesame seed hulls, more information is needed as to chemical structure and biological activities of polysaccharides.

To date, most studies of dehulled sesame meals have been concerned with oil, flavonoids, and proteins (13). The polysaccharides of sesame have received little attention (9, 14). Furthermore, there is no research about the structural characterization of sesame hull polysaccharides. Therefore, the present knowledge of structural characterization and functional properties is deficient, and further study is required.

The aim of this work was to get purified polysaccharides from sesame hull and then characterizes its primary structure. In addition, the radical-scavenging assay and ferrous ion chelating assays were also carried out. These works might provide some useful information on the structural aspects of sesame hull polysaccharides for its applied research.

## Materials and Methods

### Materials and Chemical Reagents

The seeds of sesame (*Zhengzhi 13*, white) were purchased from Henan Sesame Research Center and stored in the refrigerator (−20°C) before use. Sesame hulls were obtained according to previous reports (15, 16). The sesame seeds were soaked for 10 min in sufficient volume of distilled water at room temperature to allow the sesame skins to absorb water and swell and then knead them. The skins are broken into pieces and have broken away from the surface of the seeds, revealing the white-colored kernels. Both kernels and skins are then dried together in an oven (50°C) for 6 h. The crushed hulls shrink more and become smaller so that they can be sifted through a sieve (30 mesh) and collected. The ratio of hull mass to seed mass was between 17% and 19%, which was also the yield of hull. The dried hulls removed oil by the Soxhlet extraction method and placed in oven (50°C) for 6 h to evaporate excess solvent (16). Finally, samples were placed in desiccators before use. Furthermore, the protein content (4.41%) and ash content (33.05%) of defatted hulls were determined according to the AOCS methods (17). Potassium hydroxide, hydrochloric acid, ethanol, and toluene were analytical grade.

### Isolation and Purification of Polysaccharide

This procedure was according to the previous studies (18, 19). Briefly, the dried defatted sesame hulls (1 g) were mixed with 25 mL KOH solution (5 wt%) at 50°C in an ultrasonic extractor for 30 min. Then, put in a water bath kettle with continuous magnetic stirring at 50°C for 3 h. After that, the filtrates were obtained through a suction filter and then neutralized to pH 5.5 with hydrochloric acid (6 mol/L). Then, the mixtures were centrifuged at 5,180 *g* for 35 min by a LD 5–10 centrifugal machine (Jingli Centrifuge Co., Beijing, China). Concentrate the liquid supernatant to 1/10 of its original volume by rotary vacuum evaporator and pour in 3 times anhydrous ethanol. After centrifugation, the precipitate was resolubilized and dialyzed for several days using a dialysis bag (Solarbio, 3,500 Da), until there is no salt in the dialysate. It is then concentrated again and freeze-dried for 48 h. At this stage, the yield of polysaccharides without deproteinization was 12.10%. It was then resolubilized, and the protein was removed by the Sevag method (20) and decolored with AB-8 resin (Solarbio). The polysaccharide solution was freeze-dried and labeled as SHP. Then, it was purified by column chromatography (cellulose DEAE-52, 55 mm × 300 mm, Solarbio) which eluted in sequence with NaCl solutions (0, 0.1,0.3, 0.5, and 0.7 mol/L) at 2 mL/min. Elution curves were drawn. The collected fractions were dialyzed and lyophilized. Three purified fractions were obtained and labeled as SHP-1, SHP-2, and SHP-3. The sugar content of the purified polysaccharides was detected by using the phenol–sulfuric acid method (21).

### Monosaccharide Analysis

The monosaccharide composition was detected by the reported method (18). Briefly, samples (5.00 ± 0.05 mg) were blended with 125 μL 72% H_2_SO_4_ and 1.35 mL distilled water and then placed in a circulating hot air oven. Set the temperature to 105°C for 2.5 h. After that, the hydrolysate is neutralized and diluted 50 times with distilled water. Then, diluent was filtered through a filter (0.22 μm) and then was injected into the high-performance anion exchange chromatography (Dionex ICS-3000, Sunnyvale, CA, United States) which is equipped with CarbopacPA-20 column. The column temperature was set at 30°C, and flow rate was 0.5 mL/min; gradient elution was carried out at different times with different ratios of mobile phases: A (0.1 mol/L NaOH) and B (0.1 mol/L NaOH, 0.2 mol/L NaAc). The monosaccharide standards were also detected to make a standard curve graph. The peak areas of the corresponding compounds in the unknown samples were brought into the standard curve, and the concentrations were calculated.

### Molecular Weight Distribution

The molecular weight distributions were determined according to Guo et al. (9, 22). The HPSEC (high-pressure size exclusion chromatography, Malvern Viscotek TDA305max) is equipped with a Guard column, A6000M column, a refractive index detector (RID), and a low-angle light-scattering system (LALS). After preparation of polysaccharide solution (1 mg/mL), set parameters as follows: injection volume of 100 μL; the temperature of both column and detector temperature was 40°C; NaNO_3_ (0.1 mol/L) containing NaN_3_ (0.03 wt%) was used as the eluent solution with a flow rate of 0.7 mL/min.

### Fourier Transform Infrared Spectrometer Analyses

Dried polysaccharide samples with KBr powder were ground. The Nicolet iN10 Fourier transform infrared spectrophotometer (Thermo Nicolet Corporation, United States) was used to characterize the FTIR (Fourier transform infrared spectrometry) spectra of the polysaccharide samples. The spectra were recorded between 400 cm^–1^ and 4,000 cm^–1^ (23).

### Scanning Electron Microscopic Observations

A scanning electron microscope (SEM, Quanta 250FEG, FEI, United States) was used to characterize sample morphology according to the previous study (24). The polysaccharide sample was coated with gold, and then, images were captured using an accelerating potential of 3 kV with 1,000–5,000 × magnification under a high vacuum.

### Thermal Measurements

Thermal stability was determined according to the previous study (25). The samples (about 10 mg) were weighed and placed in a NETZSCH STA-449C thermal gravimetric analyzer and then heated at a rate of 10°C/min from 45°C to 700°C with stable nitrogen flow at 50 mL/min.

### Methylation and GC-MS of Polysaccharides

In this work, the SHP-2 was reduced to neutral polysaccharides with EDC and NaBH_4_ according to the previous report (26). The methylation analysis was according to the previous studies (22, 27) with modifications. In brief, dissolve 2 mg reduced product in DMSO by heating in an ultrasonic water bath. About 20 mg of sodium hydroxide powder was added, charged with nitrogen, and sonicated for 20 min. After cooling, methyl iodine (0.3 mL) was added to start a methylation reaction. Then, the reaction was terminated by adding sodium thiosulfate solution. The methylated polysaccharide was extracted by chloroform and then was hydrolyzed at 120°C for 4 h in 0.5 mL TFA (2 mol/L). Then, it was cool to room temperature and blow-dry with nitrogen. The hydrolysis product was reduced with NaBD_4_, acetylated with acetic anhydride/pyridine (1:1 v/v), and then finally extracted with dichloromethane. Then, the product was analyzed by the Agilent Technologies 7890B-7000C gas chromatography–mass spectrometry. The apparatus was equipped with a HP5-ms quartz capillary column, and the column size was 30 m × 0.25 mm × 0.25 μm. Experiment parameters were based on the literatures (28, 29). Set the temperature to 80°C and hold for 3 min, heat to 250°C at a rate of 10°C/min, and then hold for 10 min; carrier gas was helium (1 mL/min); injection temperature was 250°C, and injection volume was 1 μL. For the MS, electron impact ion source was used; set the temperature of ion source at 250°C, ionization energy of 70 eV, and mass range from 33 to 400 amu.

### NMR Spectroscopy Analyses

The ^1^H and ^13^C NMR spectroscopy, HSQC (heteronuclear single-quantum coherence), ^1^H-^1^H COSY (correlation spectroscopy), and HMBC (heteronuclear multiple-bond correlation) spectroscopy were observed by a Bruker ARX500 NMR spectrometer (Brucker, Rheinstetten, Germany) at 297.3 K according to previous report (28). Sample (50 mg) was dissolved in deuterated water (D_2_O, 99.9%, Sigma, United States) and freeze-dried and repeated several times to completely replace the H_2_O by D_2_O. Finally, the polysaccharide was dissolved in deuterated water (99.9%) and poured into the NMR tube. In the hydrogen spectrum, the peak of deuterated water was used to calibrate the chemical shift. For the determination of the carbon spectrum, the chemical shifts were calibrated using tetramethylsilane as an external standard. The data were analyzed by Bruker Topspin-NMR software 4.0.

### Antioxidant Activities of Polysaccharides

The antioxidant activities of sesame hull polysaccharide were investigated as reported previously with modifications (30, 31). The various concentrations of polysaccharide solutions were prepared by dissolving polysaccharide samples in ultrapure water; they were then stored at 4°C until needed.

#### Hydroxyl Radical (OH)-Scavenging Activity Assay

FeSO_4_ solution (50 μL, 1.8 mmol/L) and salicylic acid solution (50 μL, 1.8 mmol/L) were added to 50 μL polysaccharide solutions in turn. Then, 50 μL H_2_O_2_ solution (0.3%) was added. After 35 min, the absorbance of mixtures was detested at 510 nm. The same concentration of ascorbic acid (VC) solution was determined by the same method. The scavenging rate was obtained using the following formula:


(1) $$\text{⋅OH}\mathrm{scavenging}\mathrm{activity}\left(\%\right)=\frac{A_{0}-\left(A_{1}-A_{2}\right)}{A_{0}}\times 100\%$$

where A_1_ is the absorbance of the solution with the sample added, A_0_ is the absorbance without sample, and A_2_ is the absorbance of the sample solution without H_2_O_2_ solution.

#### DPPH (1,1-Diphenyl-2-Picrylhydrazy) Radical-Scavenging Activity

Polysaccharide solution (50 μL) was blended and reacted with 150 μL of 0.1 mmol/L DPPH solution for 30 min in a dark environment. A Multiskan FC automatic microplate reader (Thermo Fisher Scientific, Waltham, MA, United States) was used to obtain the absorbance. Ascorbic acid (VC) was also detected as the positive control. The scavenging rate was obtained by the following formula:


(2) $$\mathrm{DPPH}\mathrm{radical}\mathrm{scavenging}\mathrm{activity}\left(\%\right)=\frac{A_{0}-\left(A_{1}-A_{2}\right)}{A_{0}}\times 100\%$$

where A_0_ is the absorbance of the mixture of deionized water and DPPH solution, A_1_ is the absorbance of the mixture of polysaccharide solution with DPPH solution, and A_2_ is the absorbance of the mixture of polysaccharide solution with deionized water.

#### DMPD (*N*, *N*-Dimethyl-p-Phenylenediamine Dihydrochloride) Radical-Scavenging Activity

One milliliter of DMPD solution (0.1 mol/L) was prepared and added to 100 mL 0.1 mol/L acetate buffer (pH 5.25). Then, 0.2 mL of 0.05 mol/L ferric chloride solution was added, thereby creating the DMPD^⋅+^ (colored radical cation) in the solution. Polysaccharide solution (50 μL) was added to the DMPD^+^ solution (200 μL). After 10 min at 25°C, the absorbance was detected at 510 nm. The scavenging activity was calculated according to the following formula:


(3) $$\text{DMPD}\mathrm{radical}\mathrm{scavenging}\mathrm{activity}\left(\%\right)=\frac{A_{0}-\left(A_{1}-A_{2}\right)}{A_{0}}\times 100\%$$

where A_0_ is the absorbance of the mixture of acetate buffer and DMPD^⋅+^ solution, A_1_ is the absorbance of polysaccharide solution sample with DMPD^⋅+^ solution, and A_2_ is the absorbance of polysaccharide solution sample with acetate buffer.

#### Ferrous Ion (Fe^2+^) Chelating Ability

About 50 μL polysaccharide solution was prepared and mixed with 100 μL of 0.1 mmol/L FeSO_4_ solution, and then, 50 μL ferrozine solution (2.0 mmol/L) was added. The mixed solution was placed in dark environment for 10 min. The absorbance of sample was observed at 562 nm. The EDTA-2Na (Ethylene diamine tetraacetic acid disodium salt) was used as the control. The result was obtained using the following formula (4):


(4) $${\text{Fe}}^{2+}\mathrm{chelating}\mathrm{activity}\left(\%\right)=\frac{A_{0}-\left(A_{1}-A_{2}\right)}{A_{0}}\times 100\%$$

where A_0_ is the absorbance of the blank control solution, A_1_ is the absorbance of the mixture containing both reaction solution and sample, and A_2_ is the absorbance of the mixture containing both deionized water and sample.

### Statistical Analysis

The data were analyzed by SPSS Statistics 23.0. All of the data were expressed as mean ± standard deviation (SD). The significance of differences among mean comparisons was calculated using Duncan’s multiple range test with a significance level of 0.05.

## Results and Discussion

### Yield and Morphology of Purified Polysaccharide Fractions

**FIGURE 1:**
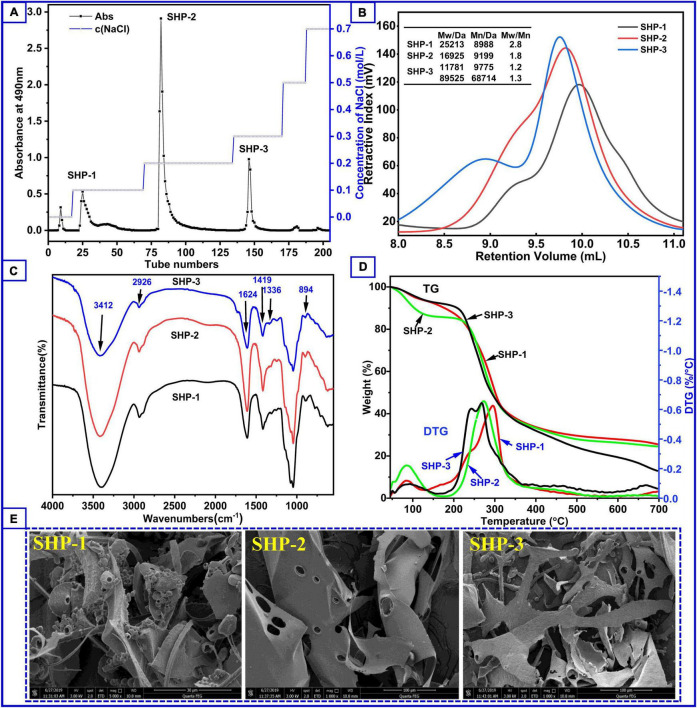
**(A)** Content of polysaccharide fractions obtained by elution at different salt concentrations during column chromatography. **(B)** Molecular weight distribution of polysaccharide samples. **(C)** Fourier transform infrared spectroscopy of polysaccharide samples. **(D)** Thermogravimetry curves (left axis) and derivative thermogravimetry curves (right axis) of SHP-1, SHP-2, and SHP-3. **(E)** The scanning electron microscope of the samples.

The yield of SHP was 6.49%, which was based on the weight of sesame hull, before fractionation by ion-exchange chromatography. In a study that also used ultrasound assistance, the yield of polysaccharides extracted from mulberry fruits was 3.13% (32). In another study, the yield polysaccharides from the *Moringa oleifera* Lam. leaf by microwave extraction was about 2.96% (33). Hence, the polysaccharide content of the sesame hull is relatively rich. After fractionation, the elution curve (Figure 1A) revealed that SHP consisted mainly of three fractions and the yield is 1.40% (SHP-1), 3.78% (SHP-2), and 1.06% (SHP-3), respectively. The total sugar content was 93.90% (SHP-1), 98.00% (SHP-2), and 91.10% (SHP-3), respectively. The peaks were clearly separated, which indicated that the three fractions had been effectively isolated from SHP. Protein content, calculated using the Bradford procedure (34), was 0.78% (SHP-1), 1.67% (SHP-2), and 1.29% (SHP-3).

Morphologies of SHP-1, SHP-2, and SHP-3 are shown in Figure 1E. Actually, the surface morphology of dried samples *via* SEM was very likely to be related to their lyophilization method or process. In this study, the apparent appearance of several purified polysaccharides being lyophilized under the same conditions is presented. SHP-1 appears very chaotic, irregular, and fragmented, with strips, sheets, curls, and an uneven surface. In contrast, SHP-2 appears as smooth, large flakes (about 100 μm) with some holes, while SHP-3 is a long strip (< 40 μm), narrower than SHP-2. The differences might be associated with different network structures and branches inside the samples (35, 36).

### Monosaccharide Composition

The sugar compositions of the purified polysaccharide fractions are shown in Table 1. The SHP-1 contained mostly galactose (41.4%), xylose (31.9 %), and arabinose (18.8%) with small amounts of glucose (2.3%), glucuronic acid (2.2%), and galacturonic acid (3.4%) units. In contrast, SHP-2 was rich in galacturonic acid (51.3%) and glucuronic acid (13.8%) together with arabinose (5.7%), galactose (4.7%), glucose (8.4%), xylose (5.9%), rhamnose (8.9%), and mannose (1.3%). SHP-3 also contained large amounts of galacturonic acid (64.1%), indicating that both SHP-2 and SHP-3 fractions were pectic-type polysaccharides (37, 38).

**TABLE 1:** Monosaccharide compositions of purified polysaccharides of sesame hulls.

Samples	Molar composition^a^ (mol%)							
	Ara^b^	Gal	Glc	Xyl	Rha	Man	GlcA	GalA
SHP-1	18.8	41.4	2.3	31.9	n.d.	n.d.	2.2	3.4
SHP-2	5.7	4.7	8.4	5.9	8.9	1.3	13.8	51.3
SHP-3	12.5	13.1	n.d.*^c^*	10.3	n.d.	n.d.	n.d.	64.1

*^a^Expressed in relative molar percentages.*

*^b^Ara, arabinose; Gal, galactose; Glc, glucose; Xyl, xylose; Fuc, fructose; Rib, ribose; Rha, Rhamnose; Man, mannose; GlcA, glucuronic acid; GalA, galacturonic acid; SHP-1, SHP-2, and SHP-3 were three purified fractions of sesame hulls polysaccharide, respectively.*

*^c^ND, not detectable.*

### Molecular Weight Distribution

The biological and chemical activities of polysaccharides are closely related to molecular weight and distribution of molecular weights; hence, understanding these parameters is necessary for assessing their potential applications in industry (39, 40). The molecular weight distribution of these samples can be seen in Figure 1B. There was one peak in SHP-1 and SHP-2 and two peaks in SHP-3. This indicates that SHP-3 polysaccharide molecules had a wide molecular weight distribution characteristic, and the homogeneity of SHP-3 was worse than other samples. Moreover, there were two components with different molecular weight in SHP-3, and weight average molecular weight (Mw) was 11,781 Da and 89,525 Da, respectively. Mw is an important data to measure chain length of polysaccharides molecules. The Mw of SHP-1 and SHP-2 was 25,213 Da and 16,925 Da, respectively. Their polydispersity indexes (Mw/Mn) were 2.805 (SHP-1) and 1.840 (SHP-2), respectively. Therefore, the polydispersity indexes of SHP-2 are smaller relative to the SHP-1, which indicates that SHP-2 has a better homogeneity than SHP-1.

### FTIR Characterization

FTIR spectroscopy is commonly used to identify characteristic organic groups and some chemical bonds of polysaccharides (41, 42). The spectra of purified polysaccharide in the range from 4,000 to 500 cm^–1^ are shown in Figure 1C. These fractions showed similarity in the main absorption spectra, but exhibited difference in peak intensity. Two absorption bands (2,800–3,500 cm^–1^ and 500–1,700 cm^–1^) were clearly observed for the three polysaccharides. The appearance of a sharp and strong peak between 3,600 and 3,200 cm^–1^ is associated with -OH stretching due to the large number of intra-molecular and inter-molecular hydrogen bonds in samples (39, 43). Peaks at 2,926 cm^–1^ are associated with asymmetric and symmetric stretching of CH_2_. Stretching peaks at around 1,624 cm^–1^ correspond to free carboxyl groups (1,700–1,600 cm^–1^), while those at around 1,336 cm^–1^ and 1,419 cm^–1^ are associated with the vibration of C-O stretching in symmetric COO- (1,300–1,400 cm^–1^) (44). The result suggests that there are uronic acid components in SHP-1, SHP-2, and SHP-3 and is in agreement with sugar composition analysis results (Table 1). The absorption band range from 1,300 to 1,000 cm^–1^ is designated as the fingerprint region and is characterized by vibrations of several types of bonds in carbohydrate rings, including C-O-C and C-O. These peaks indicate the presence of a pyran-type structure in all three fractions (45, 46). The peak at approximately 894 cm^–1^ is related to β-glycosidic linkages in polysaccharide chains, and the peak at 844 cm^–1^ can be associated with α-pyranose (22). Therefore, the FTIR spectra of these purified polysaccharide fractions all have absorption peaks typical of heteropolysaccharides.

### Thermal Analysis

Thermal stability data represent information necessary for polysaccharides’ applications in the food and biopharmaceuticals industries (23). The thermogravimetric curve can be used to analyze the weight loss and thermal behavior of samples. The DTG (derivative thermogravimetry) curve helps to determine the maximum value of a mass loss process and assists us in identifying some of the smaller mass loss processes. It can also be used in processes that indicate whether a chemical or physical reaction is taking place at a certain temperature stage. The thermogravimetric (TG) curve to temperature (or time) (Figure 1D) shows three stages of degradation. The initial stage of mass loss occurred from 45°C to 150°C and was because of the evaporation of water. The losses were 5.9% (SHP-1), 12.3% (SHP-2), and 5.6% (SHP-3). A significant peak in this temperature range can also be seen in the DTG curve, which indicates that a significant weight loss does occur. Moreover, SHP-2 volatilizes a large number of small molecules (i.e., water) compared to the other two fractions. The second stage (150–350°C) was associated with the decomposition and depolymerization of the chemical structure (9), and the fractions lost weight of 68.6% (SHP-1), 63.3% (SHP-2), and 81.6% (SHP-3). In the DTG curve, the maximum peak value represents the maximum rate of sample mass loss. Therefore, mass loss is fastest at this stage, with maximum loss rates at 294.8°C, 273.1°C, and 266.9°C for SHP-1, SHP-2, and SHP-3, respectively. Apparently, SHP-3 reached its fastest decomposition temperature much earlier. The third stage of mass loss was between 350 and 600°C, and the percentages of weight loss were found to be 25.5% (SHP-1), 24.5% (SHP-2), and 12.8% (SHP-3). The loss of quality at this stage is relatively small and might be related to the oxidation of carbonaceous organic material (16, 47). In general, if the polymers in the sample are relatively stable, then only the small molecular weight molecules are lost below 300°C in the nitrogen environment. Whereas range from 300 to 600°C, the organic polymers are mostly degraded and volatilized. Then, the stable inorganic compounds remain above 600°C. However, for the polymers containing ring and aromatic structures, they do not fully volatilize above 600°C, but form the charcoal residue. Therefore, the change in mass loss is almost minimal above 600°C, as can also be seen from the DTG curves (6, 18). By understanding the changes in these polysaccharide fractions, it is possible to provide these thermodynamic aspects for their application studies.

### Methylation and NMR Spectra Analysis

From the results of the previous work, SHP-2 is the main component of sesame hull polysaccharides. Meanwhile, it has the most homogeneous molecular weight distribution and possesses a good antioxidant activity. Therefore, a more in-depth structural characterization of SHP-2 was carried out.

The methylation analysis is the most widely method to obtain the glycosidic linkages in structural polysaccharide chemistry. It involves exhaustive methylation and hydrolysis of the polysaccharide to a mixture of monomeric methylated sugars, which are then separated, identified, and quantified. The positions of glycosidic linkages in the polysaccharide correspond to the positions of unsubstituted hydroxyl groups in these methylated monosaccharides (48, 49). The Fourier infrared spectra of SHP-2 before and after methylation can be seen in Supplementary Figure 1. After methylation treatment, the peak of FTIR spectra near 3,400 cm^–1^ is almost disappeared, which proved that the methylation was completed. The GC-MS spectra of PMAAs (partially methylated alditol acetate) for SHP-2 are shown in Supplementary Figure 2. The analysis of PMAAs was assessed using the standard database from the Complex Carbohydrate Research Center of University of Georgia combining the ionization pattern of PMAAs, and then, the result was shown in Table 2. The primary ions formed during the ionization of PMAA at EI follow a pattern: (1) The break between two methylated carbon atoms is the easiest and the resulting ions are more abundant; (2) when the break occurs between a methylated carbon atom and an acetylated carbon atom, the cationic fragment containing the methylated carbon atom is formed first; (3) the break between two acetylated carbon atoms is less likely to occur and therefore the resulting ions are less abundant. The resulting ion abundance is also very low. Besides, the groups, such the -HOAc (m/z 60), -CH_2_CO (m/z 42), and -CH_3_OH (m/z 32), break away from the primary ion to form the secondary ion with the corresponding ion abundance (28, 50). Taking 1,5-Tri-*O*-acetyl-1-deuterio-2,3,4,6-tri-*O*-methyl-D-galactitol (derived from 1-linked-D-Gal*p*) as an example, the mass fragmentation was mainly m/z 71, 87, 101, 102, 118, 129, 145, 161, 162, and 205. Similarly, all the inter-glycosidic linkage patterns were obtained as shown in the following: T-Rha*p*(1→, →4)-Xyl*p*(1→,T-Gal*p*(1→, →3,5)-Ara*f*(1→, →2)-Glc*p*(1→, →4)-Gal*p*(1→, →4)-Man*p*(1→, →6)-Glc*p*(1→ with the molar ratio of 12.2 : 4.6 : 3.2 : 4.2 : 21.1 : 42.1 : 2.9 : 9.7. The ratio was approximately consistent with the monosaccharide analysis.

**TABLE 2:** Methylation analysis result of SHP-2.

PMAAs (partially methylated alditol acetates)		Linkage patterns	Major mass fragment (*m/z*)	Retention time (min)	Relative amount /mol%
A	2,3,4-Me_2_-Rha*p*	T-Rha*p*(1→	43, 59, 72, 89, 102, 118, 131, 162, 175, 203	11.90	12.2
B	2,3-Me_2_-Xyl*p*	→4)-Xyl*p*(1→	43, 59, 71, 87, 102, 118, 129, 145, 162, 189, 207, 253	13.11	4.6
C	2,3,4,6-Me_4_-Gal*p*	T-Gal*p*(1→	43, 59, 71, 87, 102, 118, 129, 145, 161, 162, 175, 205	13.94	3.2
D	2-Me-Ara*f*	→3,5)-Ara*f*(1→	43, 59, 74, 85, 99, 118, 130, 142, 160, 207, 261	14.41	4.2
E	3,4,6-Me_3_-Glc*p*	→2)-Glc*p*(1→	43, 59, 71, 87, 101, 129, 145, 161, 174, 190, 205, 234	15.20	21.1
F	2,3,6-Me_3_-Gal*p*	→4)-Gal*p*(1→	43, 57, 71, 85, 99, 118, 129, 147, 161, 233, 281, 305	15.61	42.1
G	2,3,6-Me_3_-Man*p*	→4)-Man*p*(1→	43, 87, 99, 118, 129, 147, 173, 208, 233	15.81	2.9
H	2,3,4-Me_3_-Glc*p*	→6)-Glc*p*(1→	43, 59, 71, 87, 102, 118, 129, 143, 162, 173, 189, 233	16.51	9.7

**FIGURE 2:**
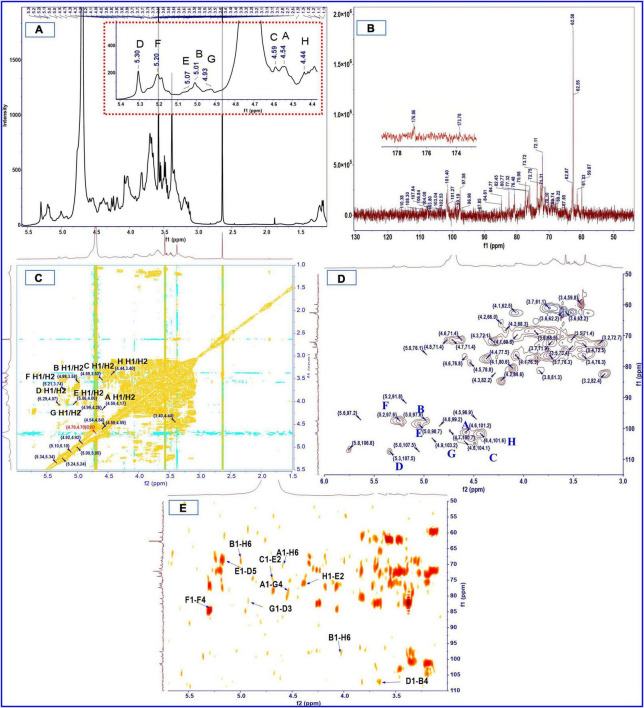
^1^H **(A)**, ^13^C **(B)**, COSY **(C)**, HSQC **(D)**, and HMBC **(E)** spectra of SHP-2.

The methylation analysis provides quantitative information but no information on the relative order of the sugar residues or on their anomeric nature. Determination of the complete structure of a polysaccharide requires complementary analyses, such as by NMR. The anomeric proton signal in low field (δ > 5.00 ppm) was generally attributed to the α-glycosidic conformation, and 4.30–5.00 ppm was assigned to β-glycosidic (49, 51). The anomeric proton signals were obtained from the ^1^H NMR spectra at δ 4.54, 4.59, 5.24, 4.44, 5.34, 5.05, 4.98, and 4.92 ppm, respectively (Figure 2A). There was no absorption in the range of 6–8 ppm, indicating that there were no ferulic acid nor phenols in SHP-2 (52). The chemical shifts of anomeric carbons occur at 90.0–110.0 ppm. The ^13^C NMR spectrum (Figure 2B) showed eight peaks: 101.3, 104.1, 97.6, 101.7, 107.5, 99.1, 97.6, and 103.3 ppm, respectively. Several typical peaks also corroborated the monosaccharide composition. The signals at 173.7 and 176.8 ppm implied the presence of glucuronic acid or galacturonic acid, respectively. There was a strong peak at 3.58 ppm in ^1^H-NMR spectra, which confirmed the existence of -O-CH_3_ group in glucuronic acid and galacturonic acid. The anomeric proton signals and carbon signals were matched one to one from the HSQC (heteronuclear single-quantum coherence) NMR spectra (Figure 2D). They were 101.3/4.54, 104.1/4.59, 97.6/5.24, 101.7/4.44, 107.5/5.34, 99.1/5.05, 97.6/4.98, and 103.3/4.92 ppm, respectively. The chemical shifts of *β-D* or α-L configurations were in the range of 100–110 ppm, the signals of α-D or β-L configuration were in the region of 90–100 ppm, and these rules can be used to determine their configurations (53, 54). Then, the analysis is continued by H, H-COSY (correlation spectroscopy) spectrum (Figure 2C) with the signal 101.3/4.54 as an example. δ 4.17 ppm can be assigned to H-2 due to its correlation with H-2 (4.54 ppm) from the COSY spectrum and published data. Similarly, the chemical shifts of H3, H4, H5, and H6 were assigned to 3.74, 3.51, 3.49, and 1.22 ppm, respectively. Then, the correlation with C-1 to C-6 can be found in the HSQC spectrum and was 101.3, 71.4, 81.1, 73.4, 72.3, and 17.4 ppm, respectively. Besides, it is also possible to verify the signal by capitalizing on two- and three-bond couplings in HBMC (Heteronuclear Multiple Bond Coherence) spectra (Figure 2E). For instance, the correlation can be found between C-2/C-3 and H-1 in one monosaccharide sugar unit or polymers. Therefore, combining the above analysis and reference (55), these chemical shifts were attributed to the T-β-L-Rha*p* (A). In the same way, through the analysis of data and references (56–63), every residue (A-H) was identified, and the chemical shifts are list in Table 3.

**TABLE 3:** Chemical shifts of resonances in the ^1^H and ^13^C NMR spectra of SHP-2.

Sugar residues		Chemical shifts (ppm)					
		C1/H1	C2/H2	C3/H3	C4/H4	C5/H5	C6/H6
A	T-β-L-Rha*p*(1→	101.3/4.54	71.4/4.17	81.1/3.74	73.4/3.51	72.3/3.49	17.4/1.22
B	→4)-α-D-Xyl*p*(1→	97.6/4.98	72.3/3.53	73.9/3.75	70.4/3.65	62.2/3.58	-
C	T-β-D-Gal*p*(1→	104.1/4.59	71.6/3.52	74.1/3.68	69.7/3.96	76.6/3.74	62.4/3.53
D	→3,5)-α-L-Ara*f*(1→	107.5/5.34	80.8/4.01	82.1/4.29	82.1/4.09	68.5/3.89	-
E	→2)-α-D-Glc*p*A(1→	99.1/5.05	76.3/4.10	72.4/3.74	72.1/4.30	71.4/4.60	173.7/-
F	→4)-α-D-Gal*p*A(1→	97.6//5.24	68.7/3.74	70.1/4.05	84.6/4.23	77.6/4.36	176.8/-
G	→4)-β-D-Man*p*(1→	103.3/4.92	71.9/4.32	74.5/3.98	78.1/4.04	76.4/3.73	62.6/4.09
H	→6)-β-D-Glc*p*(1→	101.7/4.44	72.3/3.40	76.3/3.55	69.2/3.58	76.3/3.61	69.8/4.07

The order and position of the linkage between monosaccharide residues can be further inferred from HMBC. The HMBC spectrum (Figure 2E) indicated that C-1 of residue A (T-β-L-Rha*p*) was correlated with the H-6 of residue H (1,6-linked-β-D-Glc*p*) and H-4 of residue G (1,4-linked-β-D-Man*p)*, namely A C1/H6 H; A C1/H4 G. Similarly, C-1 of residue H was correlated with the H-2 of 1, 2-linked-α-D-Glc*p*A (H C1/H2 E); C-1 of residue D corresponded to H-4 of 1,4-linked-α-D-Xyl*p* (D C1/H4 B); C-1 signal of 1, 2-linked-α-D-Glc*p*A correlates with the H-5 of 1,3,5-linked-α-L-Ara*f* (E C1/H5 D); H-6 of residue H was correlated with C-1 signal of residue B (B C1/H6 H); C-1 of residue G was corresponded to H-3 of residue D (G C1/H3 D). It is worth noting that C-1 signal of 1,4-linked-α-D-Gal*p*A correlates with the H-4 of 1,4-linked-α-D-Gal*p*A (F C1/H4 F), while C-4 of residue F correlates with the H-1 of another residue F (F C4/H1 F). No linkage signal seems to be found for residue F to other residues, so multiple residues F may be linked to each other to form a chain-like structure. In addition, a previous study of our laboratory also showed the presence of pectin polysaccharides in sesame hulls (9). Therefore, the HG (homogalacturonan) which is a linear polymer composed of 1,4-linked galacturonic acid and commonly found in pectin polysaccharides (64) might be present in SHP-2. In addition, based on the above-mentioned galacturonic acid of 51.3%, it can be inferred that HG is the dominant main component of SHP-2.

### Antioxidant Activity *in vitro*

The antioxidant ability exerted by different antioxidants requires different evaluation methods (65). Accordingly, several different methods have been used for the assessment of antioxidant ability in this study, and the results are analyzed and discussed as follows.

#### Hydroxyl Radical (⋅OH) Scavenging Activity

**FIGURE 3:**
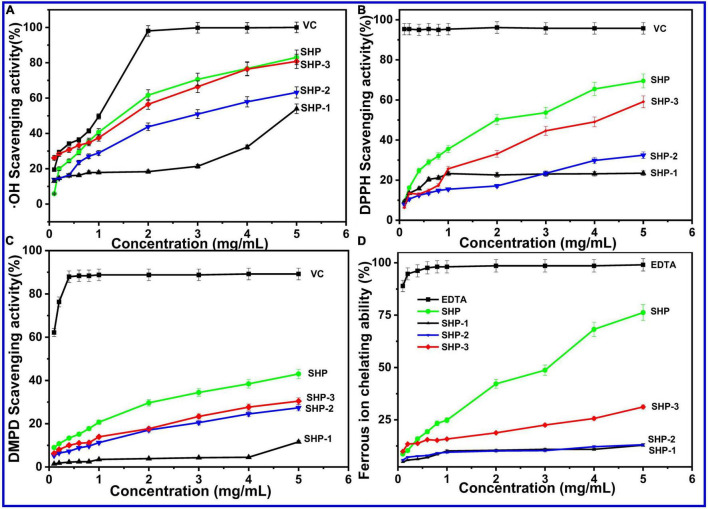
**(A)** Hydroxyl radical (⋅OH) scavenging activity. **(B)** DPPH-free radical-scavenging assay. **(C)** DMPD radical-scavenging activity. **(D)** Ferrous ion chelating ability of SHP-1, SHP-2, SHP-3, and VC.

The⋅OH can react readily association with many kinds of biomacromolecule in the body, such the lipids, proteins, and cell DNA, while excess free radicals would cause damage in tissue and even death of cell (66). Therefore, clearing excess hydroxyl radicals might be necessary to keep the human body healthy. The scavenging activity of polysaccharides is largely due to the hydrogen supplied from polysaccharides contact with radicals and stabilizing their chemical properties and then terminates the radical chain reaction of free radicals. Another possibility is that the polysaccharide combines with key radical ions in a chain reaction that causes the free radical chain to end (67). The results are presented in Figure 3A. Among all crude polysaccharide and purified fractions, the hydroxyl radical-scavenging ability can be ranked in the following order: SHP > SHP-3 > SHP-2 > SHP-1. The scavenging abilities of polysaccharide samples were lower than VC in terms of hydroxyl radical activity (*P* < 0.05). The scavenging activities were 83.06 %, 80.79 %, 63.19 %, 53.95 %, and 99.97% for the SHP, SHP-3, SHP-2, SHP-1, and ascorbic acid at a concentration of 5 mg/mL, respectively. This result demonstrated that SHP possessed the strongest scavenging activities, and SHP-3 showed the best activities among the purified fractions.

#### DPPH-Free Radical-Scavenging Assay

This experiment relies on the chromatic properties of stable radical cations and has been typically used to detect the antioxidative ability of pure or crude natural polysaccharides. The scavenging ability (Figure 3B) of SHP, SHP-1, SHP-2, SHP-3, and VC at 5.0 mg/mL had maximum values of 69.50%, 23.46%, 32.45%, 59.11%, and 95.72%, respectively. The radical-scavenging rates of SHP and SHP-3 markedly increased with increasing concentrations, while the rate of SHP-1 did not. The results indicate that the scavenging ability of DPPH-free radical for acidic polysaccharides was higher than that of neutral polysaccharides.

#### DMPD Radical-Scavenging Activity

The DMPD (*N, N dimethyl-p-phenylenediamine*) radical-scavenging activity experiment was commonly used to detect the oxidation resistance. The DMPD^⋅+^ (DMPD radical cation) with color was obtained in the presence of an oxidant. Antioxidant compounds are able to transfer the hydrogen atom to the DMPD**^⋅^**^+^, and then, the solution is discolored (65). The scavenging activity of the polysaccharides on DMPD free radicals showed a positive correlation in the concentrations of 0.1–5.0 mg/mL (Figure 3C). The scavenging abilities represented maximum values of 43.03% (SHP), 11.61% (SHP-1), 27.36% (SHP-2), 30.45% (SHP-3), and 89.19% (VC) at 5.0 mg/mL, respectively. Similar to other experiments, SHP showed the best antioxidant activity, followed by SHP-3 and SHP-2, and then SHP-1.

#### Ferrous Ion Chelating Ability

Electron transformation between Fe^2+^ and Fe^3+^ plays an essential role in physiological processes of the human body, such as enzymatic reactions in cellular metabolism and redox reactions (67, 68). However, a high level of ferrous ions (Fe^2+^) accelerates oxidative reactions, which can compromise health. Therefore, the intake of compounds with metal chelation activity is important for protecting the health of the body (65). The results of chelating activities of SHP, SHP-1, SHP-2, and SHP-3 at various concentrations were measured and are shown in Figure 3D. The Fe^2+^ chelating activity of these polysaccharides was concentration-dependent and ranges from 0.1 to 5.0 mg/mL. In that range, SHP displayed a sharp increase (from 8.62 to 76.23%) with increasing concentration, which indicates that SHP has an effective chelating Fe^2+^ capacity.

In this work, the relatively weak activity of purified polysaccharide fractions compared with SHP might be because the purification processes might have removed some molecules, such as proteins, responsible for the antioxidant activity (67). This remains to be investigated in future studies. On the contrary, the antioxidant activity of SHP-3 and SHP-2 was much greater than that of SHP-1. Some publications have underlined the significance of functional groups in the polysaccharide side chains, monosaccharide composition, and average molecular weight for antioxidant activity (67, 68). This result might be related to the high abundant uronic acid of SHP-2 and SHP-3, because a previous study found that the anticancer activity of polysaccharides increases with uronic acid content (69, 70). Besides, SHP-3 and SHP-2 contain many smaller molecular weight polysaccharide molecules. The functional sites or active functional groups might be more likely to contact with free radicals or ferrous ions in an aqueous solution and thus show better antioxidant capacity (71, 72). The natural polysaccharides of sesame seed hull have the potential to be used as an antioxidant.

## Conclusion

In this investigation, the SHP was extracted from sesame seed hull with a yield of 6.49%, and then, three fractions (SHP-1, SHP-2, and SHP-3) with molecular weights ranging from 11.7 to 89.5 kDa were obtained after exclusion ion-exchange column chromatography. SHP-2 with a yield of 3.78% was the dominant fragment in SHP. SHP-2 was the most homogeneous fraction in terms of molecular weight distribution compared to others. The galacturonic acid (51.3%) and glucuronic acid (13.8%) were found to be the major monosaccharide residues of SHP-2. Notably, the linear chain of 1,4-linked-α-D-Gal*p*A residues (linear homogalacturonan) was the dominant chemical structure presented in SHP-2 by NMR spectrum and methylation analysis. The SHP and purified fractions (SHP-3 and SHP-2) had good free radical-scavenging ability. Therefore, the polysaccharides from sesame hull had potential prospect as antioxidant. This study provided some basic chemical structure information of sesame hull polysaccharides for its application.

## Data Availability Statement

The original contributions presented in the study are included in the article/Supplementary Material, further inquiries can be directed to the corresponding author/s.

## Author Contributions

R-YZ: conceptualization, writing—original draft preparation, and methodology. J-HG: data curation and visualization. Y-LS: investigation. Y-FL: software. H-ML: writing—reviewing and editing and validation. W-XZ: validation. X-DW: supervision and writing—reviewing and editing. All authors contributed to the article and approved the submitted version.

## Conflict of Interest

The authors declare that the research was conducted in the absence of any commercial or financial relationships that could be construed as a potential conflict of interest.

## Publisher’s Note

All claims expressed in this article are solely those of the authors and do not necessarily represent those of their affiliated organizations, or those of the publisher, the editors and the reviewers. Any product that may be evaluated in this article, or claim that may be made by its manufacturer, is not guaranteed or endorsed by the publisher.
