# Corrigendum: Characterization of structure and antioxidant activity of polysaccharides from sesame seed hull

**DOI:** 10.3389/fnut.2022.992487

**Published:** 2022-07-29

**Authors:** Run-Yang Zhang, Jing-Hao Gao, Yi-Lin Shi, Yi-Fei Lan, Hua-Min Liu, Wen-Xue Zhu, Xue-De Wang

**Affiliations:** Institute of Special Oilseed Processing and Technology, College of Food Science and Technology, Henan University of Technology, Zhengzhou, China

**Keywords:** sesame seed, hull, polysaccharides, chemical structure, antioxidant activity

In the published article, there was an error in Table 2 as published. In the second column of Table 2, the names of several linkage patterns are incorrect. The corrected Table 2 and its caption “Table 2 Methylation analysis result of SHP-2” appear below.

**Table 2:** Methylation analysis result of SHP-2.

**PMAAs (partially methylated alditol acetates)**		**Linkage patterns**	**Major mass fragment (*m/z*)**	**Retention time (min)**	**Relative amount /mol%**
A	2,3,4-Me_2_-Rha*p*	T-Rha*p*(1 →	43, 59, 72, 89, 102, 118, 131, 162, 175, 203	11.90	12.2
B	2,3-Me_2_-Xyl*p*	→ 4)-Xyl*p*(1 →	43, 59, 71, 87, 102, 118, 129, 145, 162, 189, 207, 253	13.11	4.6
C	2,3,4,6-Me_4_-Gal*p*	T-Gal*p*(1 →	43, 59, 71, 87, 102, 118, 129, 145, 161, 162, 175, 205	13.94	3.2
D	2-Me-Ara*f*	→ 3,5)-Ara*f*(1 →	43, 59, 74, 85, 99, 118, 130, 142, 160, 207, 261	14.41	4.2
E	3,4,6-Me_3_-Glc*p*	→ 2)-Glc*p*(1 →	43, 59, 71, 87, 101, 129, 145, 161, 174, 190, 205, 234	15.20	21.1
F	2,3,6-Me_3_-Gal*p*	→ 4)-Gal*p*(1 →	43, 57, 71, 85, 99, 118, 129, 147, 161, 233, 281, 305	15.61	42.1
G	2,3,6-Me_3_-Man*p*	→ 4)-Man*p*(1 →	43, 87, 99, 118, 129, 147, 173, 208, 233	15.81	2.9
H	2,3,4-Me_3_-Glc*p*	→ 6)-Glc*p*(1 →	43, 59, 71, 87, 102, 118, 129, 143, 162, 173, 189, 233	16.51	9.7

The authors apologize for this error and state that this does not change the scientific conclusions of the article in any way. The original article has been updated.

## Publisher's note

All claims expressed in this article are solely those of the authors and do not necessarily represent those of their affiliated organizations, or those of the publisher, the editors and the reviewers. Any product that may be evaluated in this article, or claim that may be made by its manufacturer, is not guaranteed or endorsed by the publisher.

