# Supplementary material for: Characterization of Structure and Antioxidant Activity of Polysaccharides From Sesame Seed Hull
Source: Front Nutr. 2022 Jun 21;9:928972. doi: 10.3389/fnut.2022.928972 (PMC9253664; doi:10.3389/fnut.2022.928972)
Supplement: Supplementary file 1 [file Data_Sheet_1.DOCX]

**Supplementary materials**


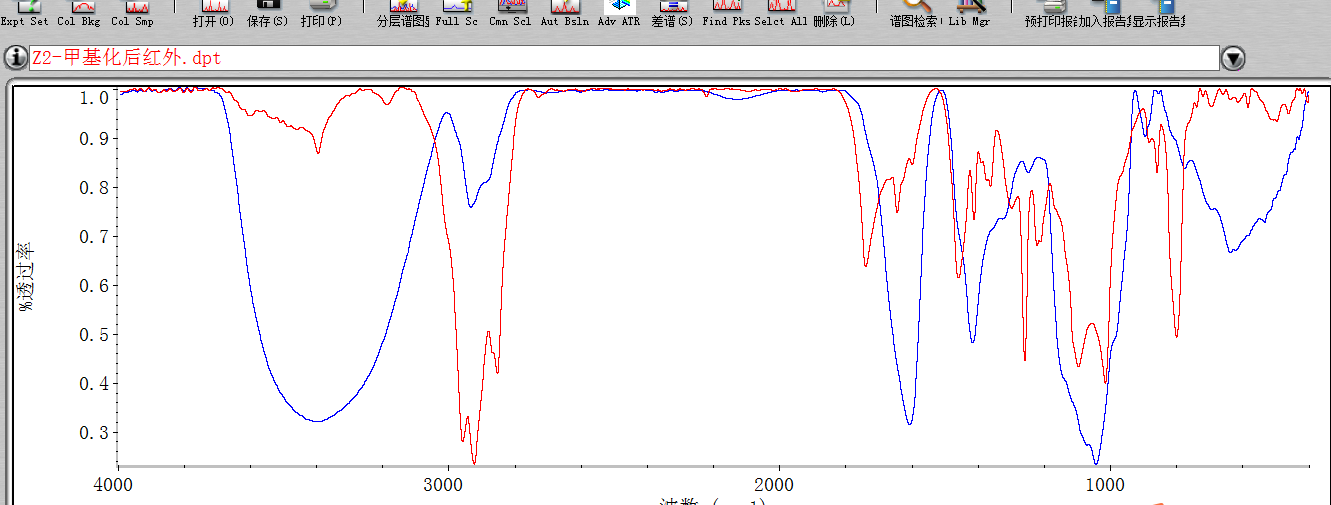


Wavenumbers (cm^-1^)

Transmittance (%)

**Fig S1. Fourier infrared spectra of sample (SHP-2) before (blue curve) and after (red curve) methylation.**

**Fig S2.** (A)


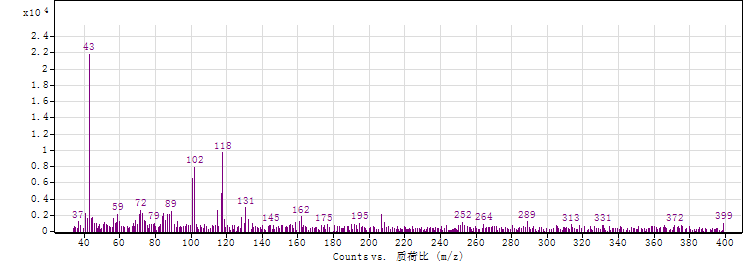


**Fig S2. (A)** **2,3,4-Me_3_-Rha*p***


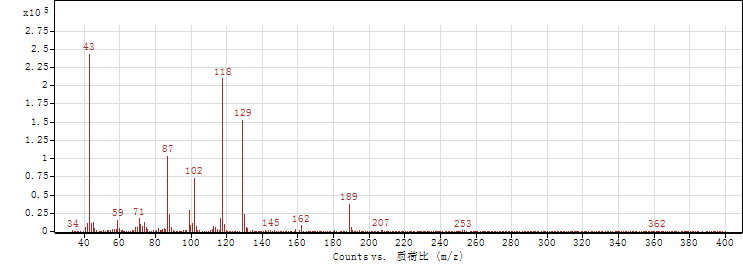


**Fig S2. (B) 2,3-Me_2_-Xyl*p***


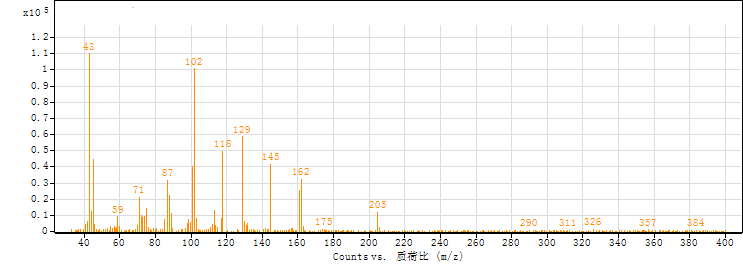


**Fig S2. (C)** **2,3,4,6-Me_4_-Gal*p***


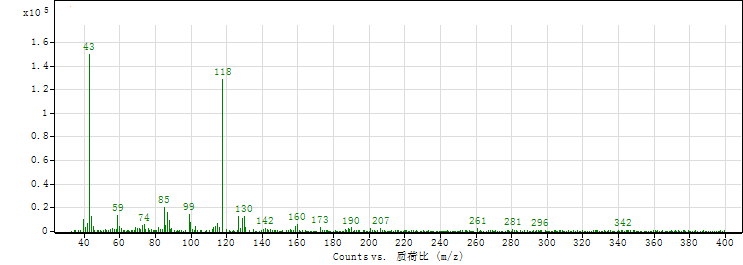


**Fig S2. (D) 2-Me-Ara*f***


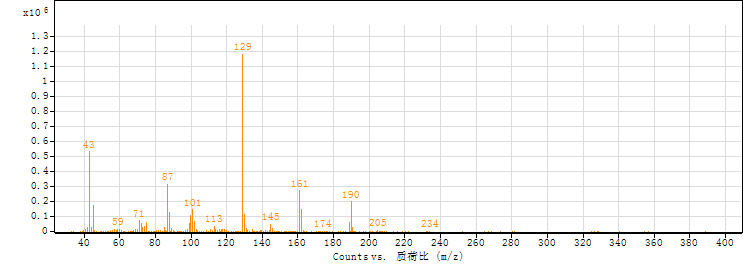


**Fig S2. (E) 3,4,6-Me_3_-Glc*p***


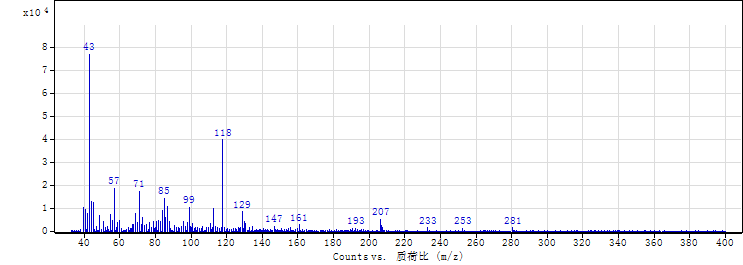


**Fig S2. (F) 2,3,6-Me_3_-Gal*p***


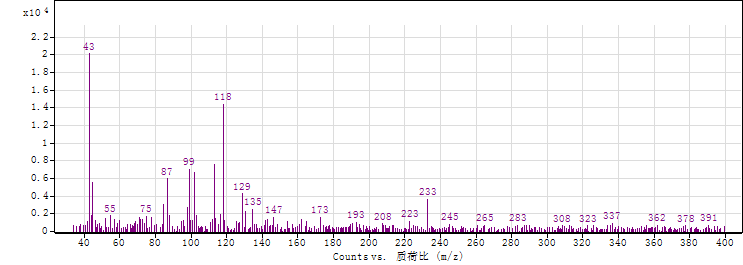


**Fig S2. (G) 2,3,6-Me_3_-Man*p***


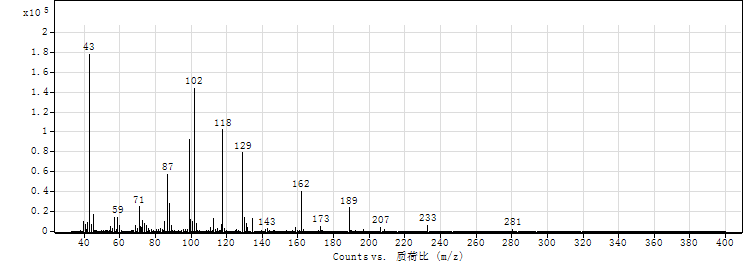


**Fig S2. (H) 2,3,4-Me_3_-Glc*p***

**Fig. S2.** **GC chromatogram (A) and corresponding mass spectra (A-H) of PMAAs for SHP-2; The other peaks may plasticizer which is a very common contaminant in methylation analysis.**
